# Editorial: Particles at Fluid Interfaces

**DOI:** 10.3389/fchem.2019.00052

**Published:** 2019-02-07

**Authors:** Erica J. Wanless, Grant B. Webber, Syuji Fujii

**Affiliations:** ^1^Priority Research Centre for Advanced Particle Processing and Transport, University of Newcastle, Callaghan, NSW, Australia; ^2^Department of Applied Chemistry, Faculty of Engineering, Osaka Institute of Technology, Osaka, Japan; ^3^Nanomaterials Microdevices Research Center, Osaka Institute of Technology, Osaka, Japan

**Keywords:** particle, colloid, interface, Pickering emulsion, foam, liquid marble

The research topic Particles at Fluid Interfaces encompasses the industrial processes and product formulations that involve the stabilization of fluid interfaces by adsorbed particles. The prevalence of these multiphase materials underpins their use in a broad range of industries from personal care and food technology to oil and mineral processing. The stabilization conferred by the adsorbed particles can be transient as found in froth flotation or long-lived as occurs within Pickering Emulsions. The particles can range in size from nanoparticles to millimeter-sized particles, and cover a spectrum from collapsed proteins, polymeric colloids of controlled size and shape to high dispersity mineral particles. In this Research Topic we present an important collection of reviews and original research articles that illustrate the pertinence of this interfacial activity in a breadth of applications backed up by fundamental investigations.

Fundamental studies provide an avenue to deepen our understanding of high interfacial area multiphase systems. In this issue a modified Langmuir trough is deployed by Yang et al. to study single air bubble interactions with a particle coated air-water interface. Atomic force microscopy is used by Xie et al. to investigate the interaction of single bubbles with the basal plane of molybdenite. McNamee et al. explore the interfacial behavior of approved food additive starch particles using their Monolayer Interaction Particle Apparatus. Meanwhile  Mitra and Evans probe the influence of temperature on dynamic contact angles of a single water droplet on a stationary brass particle backed up by computational fluid dynamics modeling. Oil foams stabilized by tetrafluoroethylene oligomer particles (Murakami et al.) and pH-responsive aqueous foams (Ito et al.) are examples of harnessing particle contact angle to control the foam stability. Controlled particle flocculation using magnetic particles as could be found in a layer of adsorbed particles on a fluid interface is modeled using Discrete Element Analysis by Neville and Moreno-Atanasio.

Particle stabilized oil-water interfaces as found in Pickering emulsions are another interface of intense current research interest and are highlighted in this special Research Topic. Xiao et al. present a review of the use of nanoparticles to stabilize such systems. A specific example of this stability is the use of nanocrystalline cellulose as reported by Varanasi et al. Pickering emulsion stability can be controlled through the use of pH-responsive latex stabilizers as demonstrated by Manga et al. Pickering stabilization using soft microgel particles is reported by Kwok and Ngai. Moreover, the transformation from an oil continuous to a water continuous phase Pickering emulsion stabilized by fumed silica upon prolonged mixing is shown by Sawiak et al. Pickering emulsions are currently favored for their prospects as delivery vehicles for therapeutics as reviewed by Tasker et al. A well-documented limitation of this encapsulation approach is the undesired leakage of the small active agents. Sun et al. chose to minimize this issue via metal coating of their cross-linked Pickering emulsion, or colloidosome for antibiotic encapsulation and delivery.

This issue also includes novel multicomponent systems that offer exciting future prospects for the deployment of particle-stabilized interfaces in formulations that frequently also contain surfactant.Whitby and Bahuon took advantage of transient droplet coalescence events to form compound droplets made of two or more drops of immiscible oils by temporarily destabilizing Pickering oil-in-water emulsions. While Borrow et al. use a high internal phase water-in-oil emulsion to selectively agglomerate fine mineral particles as a route to enhance their recovery via their adsorption to the oil-water interface. Finally, Ireland et al.; Ireland et al. outline a contactless route to liquid marble formation using electrostatic transfer of particles to pendent water droplets with a view to enhanced control in formation and prospects for scaling up production.

We believe that the Particles at Fluid Interfaces Research Topic explores the latest particle-stabilized fluid interfaces research including fundamental theory and experiments together with articles focusing on more applied research. More efficient production processes and formulations with improved control over the lifetime of product stability are two current goals in the pursuit of greater understanding. Furthermore, particle-stabilized soft dispersed systems are expected to function as a development platform for smart soft materials, and a wide range of academic and industrial future applications are anticipated. Interdisciplinary research is crucial to the continued improved utilization of particles at fluid interfaces for the development of multiphase science and engineering, as highlighted by the contributing authors to this special Research Topic who span such fields as chemistry, chemical engineering, nanotechnology, and bioresource processing and bioengineering. This Research Topic has the objective of stimulating discussion and cross-fertilization of ideas between the industries dependent upon these multiphase systems.

## Author Contributions

All authors listed have made a substantial, direct and intellectual contribution to the work, and approved it for publication.

### Conflict of Interest Statement

The authors declare that the research was conducted in the absence of any commercial or financial relationships that could be construed as a potential conflict of interest.

